# Expression Profile of Housekeeping Genes and Tissue-Specific Genes in Multiple Tissues of Pigs

**DOI:** 10.3390/ani12243539

**Published:** 2022-12-15

**Authors:** Xiangchun Pan, Jiali Cai, Yifei Wang, Dantong Xu, Yao Jiang, Wentao Gong, Yuhan Tian, Qingpeng Shen, Zhe Zhang, Xiaolong Yuan, Jiaqi Li

**Affiliations:** 1 Guangdong Provincial Key Laboratory of Agro-Animal Genomics and Molecular Breeding, National Engineering Research Center for Breeding Swine Industry, College of Animal Science, South China Agricultural University, Guangzhou 510642, China; 2 Shenzhen Branch, Guangdong Laboratory for Lingnan Modern Agriculture, Shenzhen 518120, China; 3 Genome Analysis Laboratory of the Ministry of Agriculture, Agricultural Genomics Institute at Shenzhen, Chinese Academy of Agricultural Sciences, Shenzhen 518120, China; 4 School of Veterinary and Life Sciences, Murdoch University, Murdoch 6150, Australia

**Keywords:** gene expression, housekeeping genes, tissue-specific genes, pig

## Abstract

**Simple Summary:**

Pig is an important biomedical model and a valuable source of nutrition for humans. The all-sided gene expression profile across multiple tissues engages crucial information to gain insight into their biological functions in pigs. In the present study, a comprehensive analysis has been performed on the gene expression to explore the housekeeping genes, tissue-specific genes and potentially co-expression hub genes across 14 tissues. Our results offer a fresh perspective on the transcriptome regulation of pig tissues.

**Abstract:**

Pigs have become an ideal model system for human disease research and development and an important farm animal that provides a valuable source of nutrition. To profile the all-sided gene expression and their biological functions across multiple tissues, we conducted a comprehensive analysis of gene expression on a large scale around the side of housekeeping genes (HKGs), tissue specific genes (TSGs), and the co-expressed genes in 14 various tissues. In this study, we identified 2351 HKGs and 3018 TSGs across tissues, among which 4 HKGs (*COX1, UBB, OAZ1/NPFF*) exhibited low variation and high expression levels, and 31 particular TSGs (e.g., *PDC*, *FKBP6, STAT2,* and *COL1A1*) were exclusively expressed in several tissues, including endocrine brain, ovaries, livers, backfat, jejunum, kidneys, lungs, and longissimus dorsi muscles. We also obtained 17 modules with 230 hub genes (HUBGs) by weighted gene co-expression network analysis. On the other hand, HKGs functions were enriched in the signaling pathways of the ribosome, spliceosome, thermogenesis, oxidative phosphorylation, and nucleocytoplasmic transport, which have been highly suggested to involve in the basic biological tissue activities. While TSGs were highly enriched in the signaling pathways that were involved in specific physiological processes, such as the ovarian steroidogenesis pathway in ovaries and the renin-angiotensin system pathway in kidneys. Collectively, these stable, specifical, and co-expressed genes provided useful information for the investigation of the molecular mechanism for an understanding of the genetic and biological processes of complex traits in pigs.

## 1. Introduction

Pigs are outstanding animal models for studying human diseases and developments due to their high similarities with human physiology and anatomy. In recent years, pigs have been generally used in the medical study of cardiovascular diseases [1], diabetes [2], cancer [3], and neurological disorders [4], etc. Apart from those, domestic pigs are one of the most important farm animals with a long history of breeding and great economic value. With the development of RNA sequencing, an increasing amount of data have been made publicly available around the world, including raw data containing information on various biological characteristics of different tissues and different developmental stages [5]. A better comprehension of the transcriptional profiles of different tissues in pigs will help us to leverage the pig genome for applications in various fields, such as species of evolution [6], growth and development [7], breeding [8], construction of models for a human [9], and gene-disease phenotypic association analysis [10].

According to recent reports, an increasing number of researchers have conducted and explored the RNA-seq based gene expression profiling in different mammals. For instance, Rayner et al. showed that gluteus medius and longissimus dorsi muscles were significant differences in the mRNA expression patterns in the pig [11]. Liu et al. demonstrated that thousands of genetic associations with alternative splicing and the gene expression existed in different tissues in cattle [12]. Clark et al. described that the global transcriptional features of gene expression are divided into different clusters in the sheep [13]. Therefore, we proposed that gene expression patterns in different tissues will facilitate us to elucidate the different biological functions of genes. Moreover, pig breeding programs commonly focus on the genetic improvement of reproductive traits and production, such as piglet production, the growth rate, lean meat, and others [14]. The research on the genetic mechanism of these different traits and tissues are inseparable from the transcriptional profiles of multiple tissues, such as ovaries [15], muscles [16], brains [17], and so on. Moreover, since the advent of RNA-seq, thousands of sequencing data have been generated, along with various analytical methods, to mine the information in the data [5].

Housekeeping genes (HKGs) are a class of genes that are consistently expressed in all tissues at various developmental stages and are necessary for maintaining basal cellular function [18,19]. Several efforts have attempted to define the complete set of HKGs by RNA-seq. Hounkpe et al. redefined human and mouse HKGs by mining massive RNA-seq datasets [20]. Zhang et al. found 2654 HKGs in beef cattle using large-scale RNA-seq data [21]. Joshi et al. explored HKGs from the aspect of stability and conservation using single-cell transcriptomics [22]. In contrast, the tissue-specific genes (TSGs) are generally highly expressed in certain tissues and not expressed or lowly expressed in others [23]. The tissue specificity is controlled by different regulatory procedures [24], and increasing amounts of studies have investigated the TSGs’ biological function using RNA-seq. Zhang et al. found 477 TSGs that involved in tissue differentiation and specific physiological processes across 51 tissues in the beef [21]. Sonawane et al. found that the regulation of TSGs was largely independent of the transcription factors [24]. In addition, Zhang et al. showed that TSGs evolved more slowly than HKGs [25]. Recently, the weighted gene co-expression network analysis (WGCNA) has been developed to better interpret large-scale data of RNA-seq [26]. WGCNA is often used to construct the co-expression module and identify hub genes (HUBG) within each module by describing patterns of correlation between genes based on similarities in gene expression profiles [27]. Moreover, WGCNA has been widely utilized to explore the relationship between genes and tissues for the identification of the HUBGs for various tissues in mammals, including cancer tissues in the human [28], subcutaneous adipose [29], and multiple tissues in cattle [21], brains in pigs [30], and skin and hair follicle in goats [31].

In this study, we aimed at investigate the transcriptional profiles by RNA-seq from 14 porcine tissues (backfat, gallbladder, heart, ileum, jejunum, kidney, liver, longissimus dorsi muscle, lung, muscle, ovary, endocrine brain, skeletal muscle, and spleen) to explore the association and distinctness of gene expression patterns among different tissues. Our observations will provide new insights into the biological processes of various tissues.

## 2. Materials and Methods

### 2.1. Collection of Samples

First, 609 samples were downloaded from NCBI SRA dataset including 28 samples of heart, 46 samples of the spleen, 28 samples of muscle, 63 samples of liver, 31 samples of backfat, 77 samples of lung, 16 samples of kidney, 96 samples of longissimus dorsi muscle, 91 samples of skeletal muscle, 56 samples of the ovary, 19 samples of the gallbladder, 27 samples of ileum, 21 samples of the jejunum, and 27 samples of endocrine brain (pituitary gland and pineal gland) (Table S1).

### 2.2. Data Quality Control, Alignment, and Processing

A uniform process was used to analyze each sample. The raw data of RNA-seq were processed into clean data using FASTP (0.23.2) software with default parameters for quality control [32]. The index of the reference genome was built utilizing the hisat2-build program of HISAT2 software (v2.1.0) [33]. Subsequently, the clean data of each sample were mapped to the reference genome using HISAT2 software, and the resulting SAM files were converted to BAM format using Samtools (v.1.11) [34]. The genes of each sample were assembled and quantified utilizing STRINGTIE (v.2.1.1) [35]. Furthermore, in order to unify the standard, we merged all files using the merge parameter of STRINGTIE. Finally, the corrected expression levels of genes were calculated based on fragments per kilobase of transcript per million mapped reads (FPKM). Moreover, the pigs reference genome version was Sscrofa11.1.105.

### 2.3. Gene Expression Pattern among Tissues

The gene of FPKM <1 was generally considered to be an inactive gene [36,37]. In this study, to investigate gene expression patterns of 14 tissue in pigs, we counted the number of inactive genes in each tissue and calculated the genes that FPKM >1 of at least one sample in all samples for sample clustering, and calculated the genes that expressed in at least one sample for subsequent analysis. Subsequently, the cluster of PCA was plotted with the genes that FPKM >1 of at least one sample in all samples. Finally, the genes expressed in at least two replicate samples were retained for subsequent analysis. The mean and median of gene expression values in replicate samples (the samples of the same tissue) were calculated, and the median was used for hierarchical clustering among tissues. In addition, the annotation information used the pig reference gtf file (Ensembl v105). Moreover, the heatmap of genes expression was generated using Pheatmap package of R for the demonstration of median and the mean of gene expression values.

### 2.4. Detection of Housing-Keeping Genes

The concept of housekeeping genes has been around for decades, but it is still loosely defined in detail. Here, we rigorously defined genes that showed constitutive expression in all tissues as HKGs according to previous studies, and these HKGs were so important in maintaining essential biological processes and cellular functions that research was warranted [18,21]. Specifically, the genes that averagely expressed FPKM >1 in all tissues were retained as preliminary HKGs. Furthermore, the coefficient of variation (CV) was used to evaluate the variable degree for each preliminary HKGs [38]. Specifically, CV was defined as the ratio of the standard deviation to the mean (CV = σ/μ, σ represented the standard deviation, and μ represented the mean) [21]. Subsequently, the CV was divided into three groups by the quartile of the total distribution, including lowly variable expression (CV ≤ first quartile), medium variable expression (first quartile < CV < third quartile), and highly variable expression (CV ≥ third quartile) [21]. Furthermore, the group of low variable expression HKGs was further divided into three groups according to the average expression in all tissues, including low expression (1 < FPKM ≤ 10), medium expression (10 < FPKM ≤ 50), and high expression (FPKM > 50) [21].

### 2.5. Detection of Tissue-Specifc Gene

To identify the TSGs, the stringent standards as previously described were used [23], including three criteria: (1) the genes of the average expression level of the top 25% in each tissue; (2) the genes of greater than 50% of the sum of those genes in all other tissues; and (3) the genes of greater than three times of expression of those genes in any other tissue were last considered as candidate genes [21,23]. Although TSG was obtained after screening, some genes were slightly expressed in non-specific tissues, then we narrowed target TSGs more rigorously so that it was expressed only in a specific tissue. In brief, the gene of TSGs that was expressed only in one tissue was screened as particular TSGs. Furthermore, the particular TSGs of the top 19 were shown in the heatmap.

### 2.6. Co-Expression Network Analysis

For the co-expression of genes among tissues, we first remained the genes that perform well in expression (FPKM >1 in all samples of each tissue), and further conducted co-expression network analysis using the weighted Gene co-expression Network Analysis (WGCNA). Moreover, the hclust function with the average agglomeration method of WGCNA package was used for cluster analysis. The soft threshold (β) was determined based on the scale-free distribution by the function of pickSoftThreshold of WGCNA. After determining the empirical power value, a one-step method was used to construct the network and detection of modules. As in the previous studies, we set parameter minModuleSize to 30 and parameter mergeCutHeight to 0.25 [21]. Subsequently, the information on tissues was used as phenotypic data and was associated with each module to look for important relationships between the modules and tissues. In this study, the module with a correlation coefficient larger than 0.65 was considered the important tissue-specific module as in previous research [21]. In addition, the important tissue-specific modules were used to subsequently analyze to investigate the core genes in the co-expression network. Briefly, the HUBGs were observed using module membership (MM) and gene significance (GS) methods [27,39], and the final determinations of HUBGs were based on |GS| > 0.2 and |MM| > 0.8.

### 2.7. Functional Enrichment and Protein–Protein Interaction Analysis and the Validation of HKGs and TSGs

In this study, the Kyoto Encyclopedia of Genes and Genomes (KEGG) and Gene Ontology (GO) analysis were conducted to investigate the function of genes by clusterProfiler package of R software. In addition, the correlation between pairwise pairs in this study was based on Pearson’s correlation coefficient. Moreover, the protein-protein interaction (PPI) analysis was conducted based on the STRING database [40] and further performed a network utilizing Cytoscape software (v3.4.0) [41]. Recently, Teng et al. presented results of PigGTEx from more than 9000 RNA-seq and built a website that could query the expression of each gene in each tissue of pig [42]. To demonstrate the reliability of our results, we used the results in the PigGTEx release V0 (http://piggtex.farmgtex.org/, accessed on 29 November 2022) to verify our HKGs and TSGs. For HKGs, we randomly selected three genes with high variation, middle variation, and low variation, and calculate the CV values using the expression in the PigGTEx for validation. For particular TSGs, if the expression level of the gene was significantly located in the top five tissues in the PigGTEx, it was considered a “pass” gene, and was shown in the supplementary table.

## 3. Results

### 3.1. Detection of Genes in Multiple Tissues

**Figure 1:**
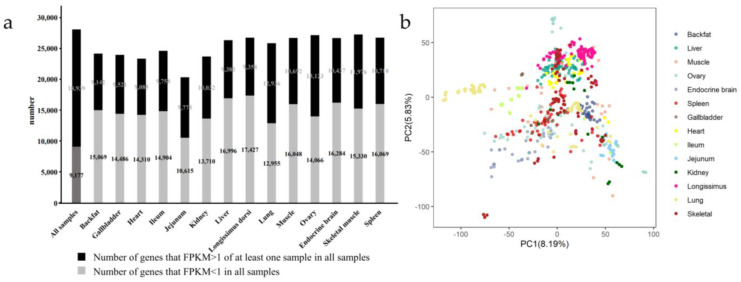

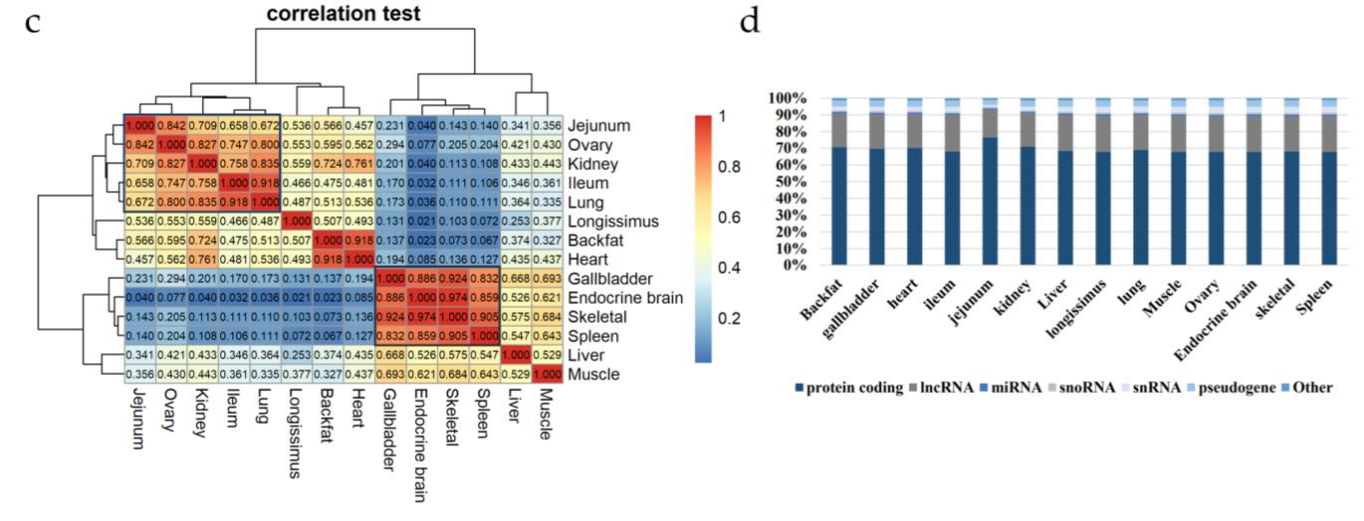
Overview of the genes by RNA-seq analyses in pigs. (**a**) The number of all detected genes and the numbers of genes detected in at least one sample with FPKM ≥1; (**b**) PCA was performed for all tissues based on the expression of genes that were detected in at least one sample with FPKM ≥1; (**c**) The heatmap of unbiased hierarchical clustering based on Pearson’s correlation coefficient for genes that detected in at least two samples. Red indicates high correlation and blue indicates low correlation; (**d**) The gene classification of genes expressed in at least two samples from each tissue.

We collected a large-scale gene expression profile covering 14 types of tissues (backfat, gallbladder, heart, ileum, jejunum, kidney, liver, longissimus dorsi muscle, lung, muscle, ovary, endocrine brain, skeletal muscle, and spleen) of pigs with a total of 609 RNA-seq samples from the public database (Table S1, see Section 2 for detail). To study the diversity and biological logical clustering across multiple tissues in pigs, we performed analysis from diverse perspectives utilizing the above transcriptome data. In total, 28,116 genes were detected in all tissues, of which 18,939 genes were detected in at least one sample with FPKM ≥ 1, which means that 32.64% (9177/28,116) were inactive genes (FPKM < 1). Respectively, 62.24% (15,069/24,211), 60.34% (14,486/24,007), 61.17% (14,310/23,395), 60.43% (14,904/24,662), 52.06% (10,615/20,390), 57.75% (13,710/23,742), 64.43% (16,996/26,379), 65.07% (17,427/26,781), 50.04% (12,955/25,891), 60.02% (16,048/26,740), 51.73% (14,066/27,189), 60.94% (16,248/26,721), 56.14% (15,330/27,306), and 60.01% (16,069/26,779) inactive genes existed in each sample of backfat, gallbladders, hearts, ileum, jejunum, kidneys, livers, longissimus dorsi muscles, lungs, muscles, ovaries, endocrine brain, skeletal muscles, and spleens (Figure 1a, Table S2). These tissues were clustered well based on gene expressions (Figure 1b).

Furthermore, we detected only 27 genes were expressed greater than 1 (FPKM ≥ 1) in all 609 samples. For each tissue, the expression level of at least 154 genes and, at most, 2698 genes were greater than 1 (FPKM ≥ 1) in all samples (Table S2). Of all the 609 samples, a total of 27,950 genes were expressed in at least two samples. For each tissue, 22,550, 22,617, 21,546, 23,087, 18,318, 21,669, 25,055, 25,996, 24,494, 25,905, 26,568, 26,060, 26,815, and 26,051 genes were detected in at least two samples (Table S2). Notably, the jejunum, ovary, kidney, ileum, lung, and longissimus dorsi muscle performed more closer association (Figure 1c). According to the annotation information, all genes were divided into seven categories, including nearly 70% of protein-coding genes and about 20% of lncRNAs (Figure 1d, Table S3). The expression of these genes seemed to show stable and specific patterns (Figure S1), therefore, we subsequently conducted a corresponding analysis.

### 3.2. The Profiles of Housekeeping Gene across Pig Tissues

**Figure 2:**
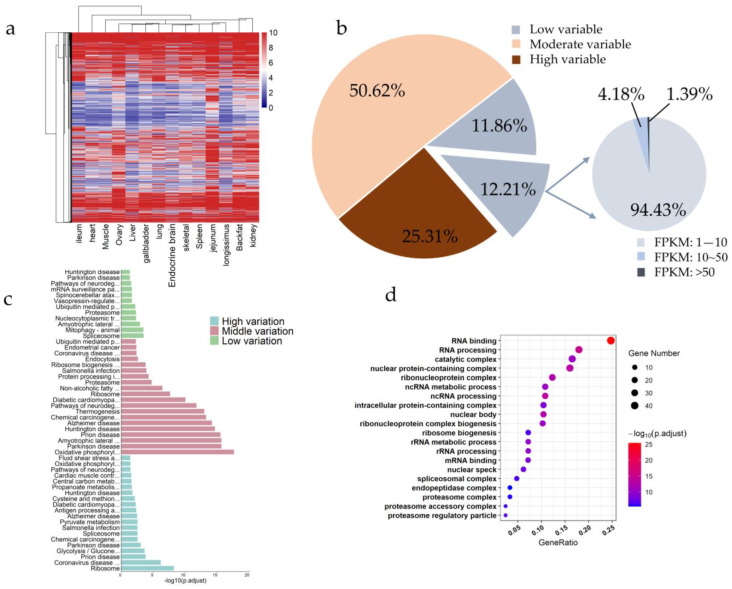
HKGs across 14 tissues of pigs. (**a**) The heatmap of preliminary screening of HKGs using the average expression in each tissue; (**b**) The percent of low, moderate, and high expression variability HKGs, and the percentage of the low, medium, and high expression levels of HKGs with low expression variability; (**c**) The KEGG analysis of preliminarily screened HKGs; (**d**) The GO analysis of low-variated HKG.

To explore HKGs in pigs, we define constitutively expressed genes as preliminary HKGs as described by Zhang et al. [21] (see Materials and methods for detail). A total of 2351 preliminary HKGs were detected to be constitutively expressed across 14 tissues, and they generally showed a relatively conservative expression pattern, but also exhibited varied expression levels among tissues (Figure 2a). Consequently, we used the coefficient of variation (CV) to quantify the expression variability of these genes across tissues (see Materials and methods for detail). According to the CV, the preliminarily screened HKGs were classified into three groups with low (0.46 which was the first quartile), medium, and high expression variability (0.78 which was the third quartile) (Figure 2b, Table S4). Furthermore, the 12.21% of HKGs which were low expression variability were further divided into three groups with low expression (1 ≤ FPKM ≤ 10), medium expression (10 < FPKM ≤ 50), and high expression (FPKM > 50). Specifically, 24.07% of HKGs were low variant, and 12.21% of these low variant HKGs were divided into 94.43% low expression level, 4.18% moderate expression level, and 1.39% high expression level (Figure 2b and Figure S2a, and Table S5). Ultimately, four HKGs (*COX1, UBB, OAZ1,* and *NPFF*) with low expression variability and high expression levels across all tissues were obtained, and the expression of low variated HKGs was shown in Table S5.

In addition, the function enrichments with the preliminary HKGs and the lowly variated HKGs were shown in Figure 2c,d, and Tables S6 and S7. We found that the ribosome, spliceosome, cellular senescence, carbon metabolism, and some metabolism signaling pathway were enriched in the high expression variability HKGs; thermogenesis, oxidative phosphorylation, ribosome, endocytosis, and proteasome signaling pathway were enriched in the medium expression variability HKGs; spliceosome, nucleocytoplasmic transport, ubiquitin mediated proteolysis, mitophagy, mRNA surveillance, proteasome, and protein export signaling pathway were enriched in the low expression variability HKGs (Table S6). Moreover, GO analysis showed that HKGs were regulated in categories including RNA binding, RNA processing, catalytic complex, ribosome biogenesis, spliceosomal complex, and so on (Figure 2d and Figure S2b–d, and Table S7). The PigGTEx was used to verify the accuracy of these HKGs (see Materials and methods for detail). We found that the expression trend of HKGs consisted with that of corresponding genes in the PigGTEx. For instance, high variated HKGs (*CTSH*, *DHRS4*, and *LYAR*) were expressed in all tissues, but the expression levels in each tissue were different, and the degree of variation were large, and the CV values were 2.85, 3.64, and 3.80, respectively. Similarly, middle variated HKGs *UGGT1* (CV = 2.18)*, SNX18* (CV = 2.56)*,* and *PLBD2* (CV = 2.41), and low variated HKGs *UBB* (CV = 1.20)*, OAZ1* (CV = 1.41)*,* and *COX1* (CV = 1.72) were stably expressed in all tissues (Figure S3).

### 3.3. Tissue-Specific Genes Expression Patterns across Pig Tissues

**Figure 3:**
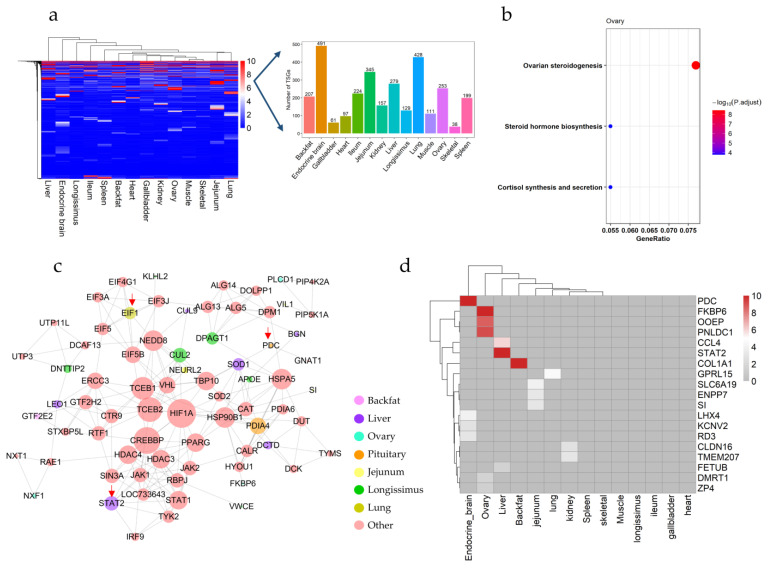
TSGs across 14 tissues of pigs. (**a**) The heatmap and number of TSGs in 14 tissues. (**b**) The KEGG of TSGs in ovaries; (**c**) The network of 74 particular TSGs based on the STRING database. Different tissues were shown in different colors, and the pink represented other non-tissue-specific genes in the network; (**d**) Heatmap of the particular TSGs with higher expression levels.

To investigate the specific genes in each tissue, we set up three layers of stringent criteria according to the previous study [21,23]. 3018 genes were left as the putative TSGs, of which 207, 61, 97, 224, 345, 157, 279, 129, 428, 111, 253, 491, 38, and 199 TSGs were in backfat, gallbladders, hearts, ileum, jejunum, kidneys, livers, longissimus dorsi muscles, lungs, muscles, ovaries, endocrine brain, skeletal muscles, and spleens, respectively (Figure 3a). Additionally, the KEGG analysis and GO analysis were applied to perform the TSGs on each tissue. As expected, TSGs in each tissue were significantly enriched in the corresponding functional pathways, which was related to their corresponding biological functions (Tables S8 and S9, and Figures S4 and S5). For example, ovarian steroidogenesis, steroid hormone biosynthesis, cortisol synthesis, and secretion signaling pathway were enriched in the ovarian tissues (Figure 3b). The signaling pathways of the renin-angiotensin system and steroid biosynthesis were enriched in the kidneys, and the signaling pathways of heart development and blood circulation were enriched in the heart (Figure S4). The reproductive process was enriched in the ovaries, and the defense response function was enriched in spleens (Figure S5).

In addition, to better investigate the TSGs across tissues, we narrowed the TSGs to thoroughly specifically expressed genes in each tissue. We defined the TSGs expressed in only one tissue as particular TSG (see Materials and methods for detail), and eventually found 74 particular TSGs (Table S10). A PPI network based on the above particular TSGs was further established utilizing the STRING database. Finally, we found that particular TSGs of the liver seemed to be the most widely connected across tissues, such as *SOD1* in livers associated with *PDIA4* in endocrine brain, and *BGN* in livers associated with *APOE* in ovaries, and *LEO1* in livers associated with *DNTTIP2* in longissimus dorsi muscles (Figure 3c). Moreover, the heatmap showed the top 19 particular TSGs. Notably, *PDC* in endocrine brain, *FKBP6*, *OOEP*, and *PNLDC1* in ovaries, *CCL4*, *STAT2* in livers, *COL1A1* in backfat, *GPRL15* in lungs showed the highly expressed level (Figure 3d), of which *PDC* and *STAT2* connected with other genes in PPI (Figure 3c). In addition, we verified the accuracy of these particular TSGs by PigGTEx (see Materials and methods for detail). Finally, the expression trends of 31 TSGs (42%) were consistent with that of the PigGTEx. For example, *CLDN16*, *TMEM207*, *ENPP7*, *TMEM38A*, *FETUB,* and *ENSSSCG00000043688* were exclusively expressed in kidney, jejunum, muscle, and liver, respectively (Table S10 and Figure S6).

### 3.4. Co-Expression Gene Network across Pigs Tissues

**Figure 4:**
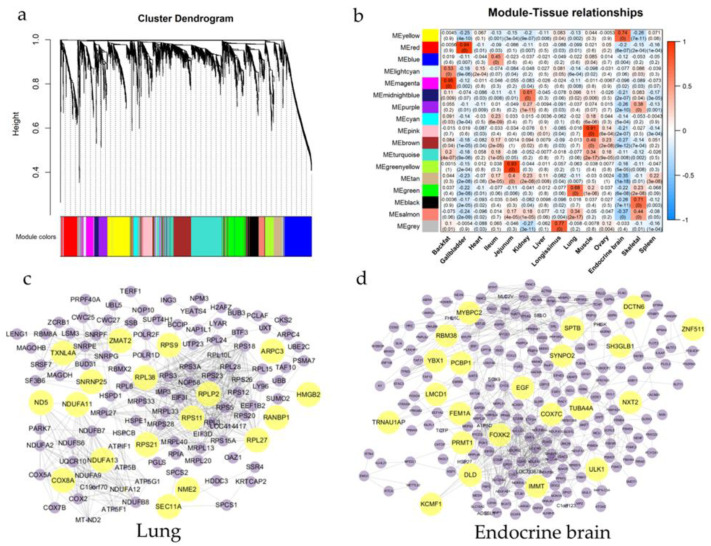
The communication network of genes across 14 tissues of pigs. (**a**) The cluster dendrogram of filtered genes with FPMK >1; (**b**) The heatmap of relationships between 17 modules and 14 tissues. The red represented a high correlation. (**c**,**d**) The networks of interactions among genes were highly correlated with traits based on the STRING database. The yellow represented HUGBs, and the purple represented non-HUGBs.

To explore the biological relationships and potential functions of core genes among tissues, the WGCNA analysis was conducted on filtered genes (FPKM >1 in all samples of each tissue) (Table S2). We found that a soft threshold for the first β was five when the *R^2^* > 0.8, meaning that the value can be used to build the scale-free network efficiently (Figure S7). Subsequently, the hierarchical clustering was constructed based on the topological overlap matrix (Figure 4a), and 17 modules were obtained (Table S11). Moreover, the associations between 17 modules and 14 tissues were shown in Figure 4b, of which eight module-tissue relationships were tissue-specific modules (*r* > 0.65, *p* value = 0). For example, the red module was highly correlated with gallbladder (*r* = 0.94, *p* value = 0), and the pink module was highly correlated with muscle (*r* = 0.91, *p* value = 0), and the magenta was highly correlated with backfat (*r* = 0.95, *p* value = 0) (Figure 4b).

Furthermore, for these eight tissue-specific modules, genes with |GS| >0.2 and |MM| >0.8 were screened as HUBGs by MM and GS methods (see Materials and methods for detail). Finally, 27, 60, 36, 0, 27, 31, 13, and 36 HUBGs were respectively observed in backfat-magenta, gallbladder-red, jejunum-green-yellow, longissimus dorsi muscles-grey, lung-grey, muscle-pink, skeletal-black, and endocrine brain-yellow modules (Table S12). The STRING networks of genes in modules showed that most genes were connected by HUBGs, meaning that these HUBGs played a central connectivity role in the genes associated with the tissues (Figure S8). For instance, the HUBGs (*ND5*, *NDUFA11*, *COX8A*, *RPS21*, *RPS11*, etc.) were widely distributed and connected to other non-HUBGs in the lungs, and the HUBGs (*EGF*, *COX7C*, *KCMF1*, *ULK1*, etc.) were widely distributed and connected to other non-HUBGs in endocrine brain (Figure 4c,d).

## 4. Discussion

Pigs are one of the first animals domesticated by humans, which has played an important role in an agricultural society and are also suitable animal models to study animal domestication and human diseases [43]. With the development of RNA-seq and analysis algorithm, the researchers on the regulations of genes expressions in multiple tissues of animals have made small progress year by year. However, the pattern of gene expression based on large-scale data from diverse tissues remain unclear in pigs. A comprehensive investigation of gene expression patterns across tissues can provide valuable insights into the biological function and regulation mechanisms underlying genetic variation in complex traits [44]. In addition, Teng et al. showed that the difference in tissue was greater than that in breed [42], thus the differences of the breed were not considered in this study. Our study focused on exploring the HKGs, TSGs, and HUBGs in 14 tissues of pigs. A total of 2351 HKGs were preliminarily identified, of which 4 HKGs were low expression variability and highly expressed HKGs. Meanwhile, a total of 3018 TSGs were detected, of which 74 TSGs were particular TSGs that were expressed in only one tissue. Moreover, 17 modules with 230 HUBGs were obtained.

Previous studies have shown that the genome-wide expression landscape of genes varies among tissues [45]. Jiang et al. observed several TSGs and HKGs in 13 tissues of multiple mammals, and TSGs were less conserved than HKGs [46]. Summers et al. showed a co-expressed atlas using 208 samples of liver nervous system tissues from pigs [47]. Yang et al. demonstrated that domestic pigs were mature early and possess higher reproductive capacity compared to wild pigs by the analysis of gene expression in brain tissues [48]. These observations indicated that differences in gene expression underlying the important traits might be related to breeding and development. In this study, we first detected the genes in each sample and observed the number of genes in the jejunum was the lowest compared to other tissues (Figure 1a). We further found that some tissues performed close association (Figure 1c). This seems to be similar to the investigation of the human Genotype-Tissue Expression (GTEx) Consortium, which found that the ovaries and livers have similar genetic backgrounds [49]. Phenotypically, a case described that cancers originally in the jejunum were misdiagnosed as originating in the ovaries [50]. Notably, we observed a high correlation between gene expression in the jejunum and gene expression in the ovaries (Figure 1c). Further investigations were needed to identify the links in these tissues. In addition, we found 27,950 genes expressed in at least two samples in 14 tissues, and the proportion of protein-coding genes in the jejunum was highest compared to other tissues (Figure 1d). Previous studies have shown that DNA damage, apoptosis, and endothelial dysfunction are greater in the jejunum than in other tissues exposed to radiation [51]. Zhang et al. showed that the expression of genes in jejunum from weaned piglets were distinct in response to different treatment [52]. Understanding gene expression patterns in different tissues including jejunum and ovary could contribute to pig breeding.

Subsequently, we uncovered 2351 HKGs of low, medium, and high expression variability, which were enriched in several key pathways that could drive the expression of genes across tissues. Notably, four HKGs *(COX1*, *UBB*, *OAZ1*, and *NPFF*) were lowly variable and highly expressed in all tissues (Figure 2). All these genes were protein-coding genes, of which *COX1* was reported that could regulate energy production in mitochondria and its impairment could result in reactive oxygen intermediates promoting oxidative stress [53], and *UBB* was an extrinsic substrate of the proteasome-dependent ubiquitin-fusion degradation pathway [54], and *OAZI* was demonstrated that its activity was associated with mitochondria [55], and *NPFF* was described that NPFF receptors were important for pubertal onset in pigs [56]. In addition, the signaling pathways of HKGs were investigated and found to be associated with various biological processes that sustain the basic cellular functions of life, including ribosome [57], spliceosome [58], thermogenesis [59], oxidative phosphorylation [60], and nucleocytoplasmic transport [61]. These results were consistent with previous reports [62]. Then, we screened low variation HKGs and placed their expression matrix in the attached table for subsequent researchers’ experimental reference (Table S5). Furthermore, 3019 genes seemed like putative TSGs after a rigorous screening, of which 74 TSGs were only expressed in one tissue (Figure 3). These putative TSGs were enriched in signing pathways related to corresponding tissue development or corresponding functions of each tissue. For instance, ovarian steroidogenesis and steroid hormone biosynthesis were reported that could be involved in the regulation of ovarian function in puberty [63,64]. Interestingly, our HKGs expressions were consistent with the PigGTEx, meaning that these results could be available reference for further research. Although only 42% of particular TSGs expression trends were consistent with PigGTEx, we found that the expression levels of TSG that did not conform to the trend varied significantly within the tissues, or these TSG were highly expressed in those tissues that we did not detect. It is possible that these results were influenced by the sample size. Another possibility was that the results were affected by the batch effect, and we strongly recommend that future studies resolve the batch effect first.

To better understand the genetic and biological processes of complex traits, we conducted the analysis of PPI and co-expression network. Remarkably, *SOD1* deficiency affected liver metabolism [65], and *SOD1* was only expressed in livers and associated with *LEO1* of endocrine brain in this study, indicating that liver metabolism might be regulated endocrine brain through the associated tissue-specific genes. *BNG* was demonstrated that related to production in mice, and *BNG* was only expressed in livers and associated with *APOE* of the ovary in this research, suggesting that ovary production might be regulated liver by the associated TSGs. Moreover, we found that *PDC* was exclusively expressed in livers, and *STAT2* was exclusively expressed in endocrine brain, and these two genes were related to each other (Figure 2c,d). In contrast, the previous report had shown that *PDC* was expressed in multiple tissues as a ubiquitous G-protein regulator [66], and this intriguing result could be due to the too-strict criteria for screening TSGs. Furthermore, we constructed 17 co-expression modules with genes from 14 tissues using the WGCNA method to identify hub genes of each tissue, and finally found a dozen of HUBGs in lungs, endocrine brain, backfat, gallbladders, jejunum, muscles, and skeletal muscles, respectively (Figure 4). Thereinto, *NDUFB9* as an important gene in the respiratory body was involved in the mitochondrial respiration of mammals [67]. *RPS11* was differentially expressed in the trachea when pigs are exposed to NH3 [68]. *EGF* was differentially expressed in swine testicular cells after heat stress [69]. *ULK1* could be activated when *PIK3C3* was overexpressed in livers [70]. Our results provide fresh insight into the association between genes of various tissues in pigs, but the underlying mechanisms in comprehensive of the HKGs, TSGs, and HUBGs among tissues in pigs still require careful elucidation and verification.

## 5. Conclusions

In this study, the transcriptomic profiles of 14 pig tissues were explored. We found that the number of expressed protein-coding genes in the jejunum was the highest. A total of 2351 HKGs and 3018 TSGs were identified, of which 4 HKGs were low variation and high expression, and 31 TSGs exclusively expressed in one tissue, respectively. Furthermore, 17 modules with 230 HUBGs were obtained. The signaling pathways of ribosome, spliceosome, thermogenesis, oxidative phosphorylation, and nucleocytoplasmic transport were enriched by HKGs, implying HKGs involved in the basic biological activities of cells. While the TSGs signaling pathways revealed that the exclusively expressed genes were likely to control the specific physiological processes of certain tissues. These results provided useful information for the investigations on the biological development and processes of various tissues in pigs.

## Data Availability

The data presented in this study are available in this article (Table S1).

## Supplementary Materials

The following supporting information can be downloaded at: https://www.mdpi.com/article/10.3390/ani12243539/s1, Figure S1: The heatmap of genes expression detected in at least two samples; Figure S2: Analysis of HKGs; Figure S3: The expression of nine randomly selected HKGs in PigGTEx; Figure S4: KEGG analysis of TSGs in each tissue; Figure S5: GO analysis of TSGs in each tissue; Figure S6: The expression of particular TSGs in PigGTEx; Figure S7: The weighted gene correlation network analysis; Figure S8: The network of interactions between genes highly correlated with trait including; Table S1: The summary of download RNA-seq; Table S2: The summary of genes; Table S3: The class of genes that expressed in at least two samples; Table S4: The CV value of HKGs; Table S5: The expression of low variated HKGs; Table S6: KEGG of HKGs; Table S7: GO of HKGs; Table S8: KEGG of TSGs; Table S9: GO of TSGs; Table S10: TSGs expressed in only one tissue; Table S11: genes in 17 modules; Table S12: HUBGs in a highly correlated module.
