# Supplementary material for: Expression Profile of Housekeeping Genes and Tissue-Specific Genes in Multiple Tissues of Pigs
Source: Animals (Basel). 2022 Dec 15;12(24):3539. doi: 10.3390/ani12243539 (PMC9774903; doi:10.3390/ani12243539)
Supplement: Supplementary file 1 [file animals-12-03539-s001.zip › supplementary figure.pdf]

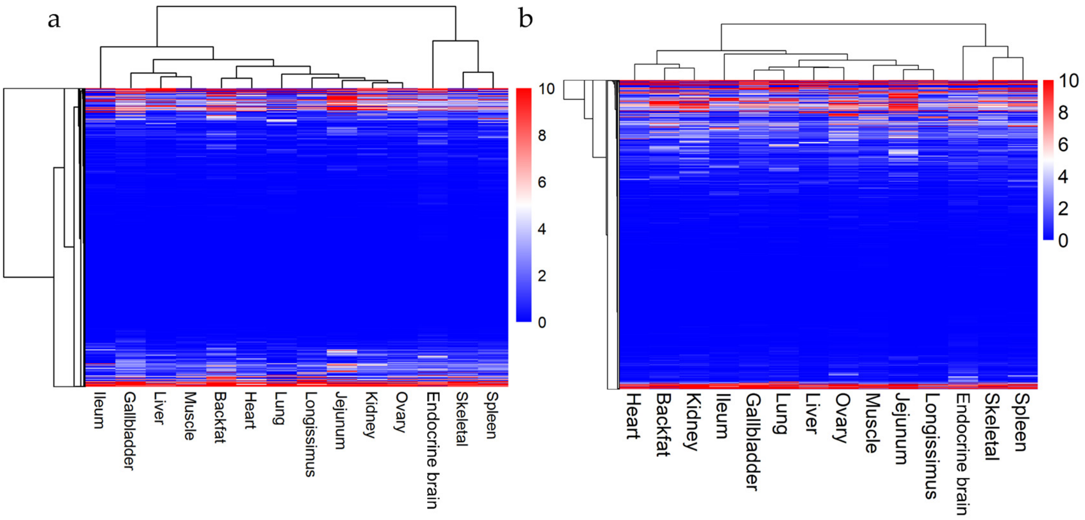

Figure S1. The heatmaps of gene expression detected in at least two samples. (a) The median of genes expressed in at least two samples; (b) The average of genes expressed in at least two samples.

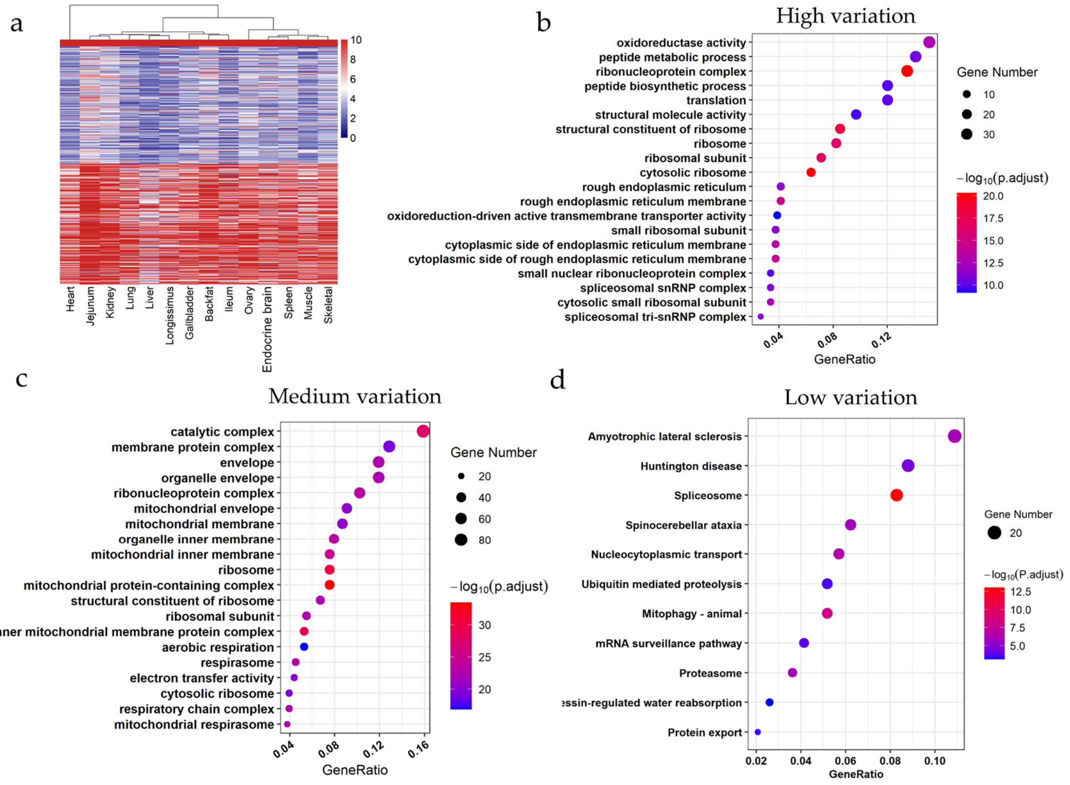

Figure S2. Analysis of HKGs. (a) Gene expression heat map of low variant HKGs; (b) The GO analysis of high variant HKGs; (c) The GO analysis of medium variant HKGs; (d) The KEGG analysis of low variant HKGs.

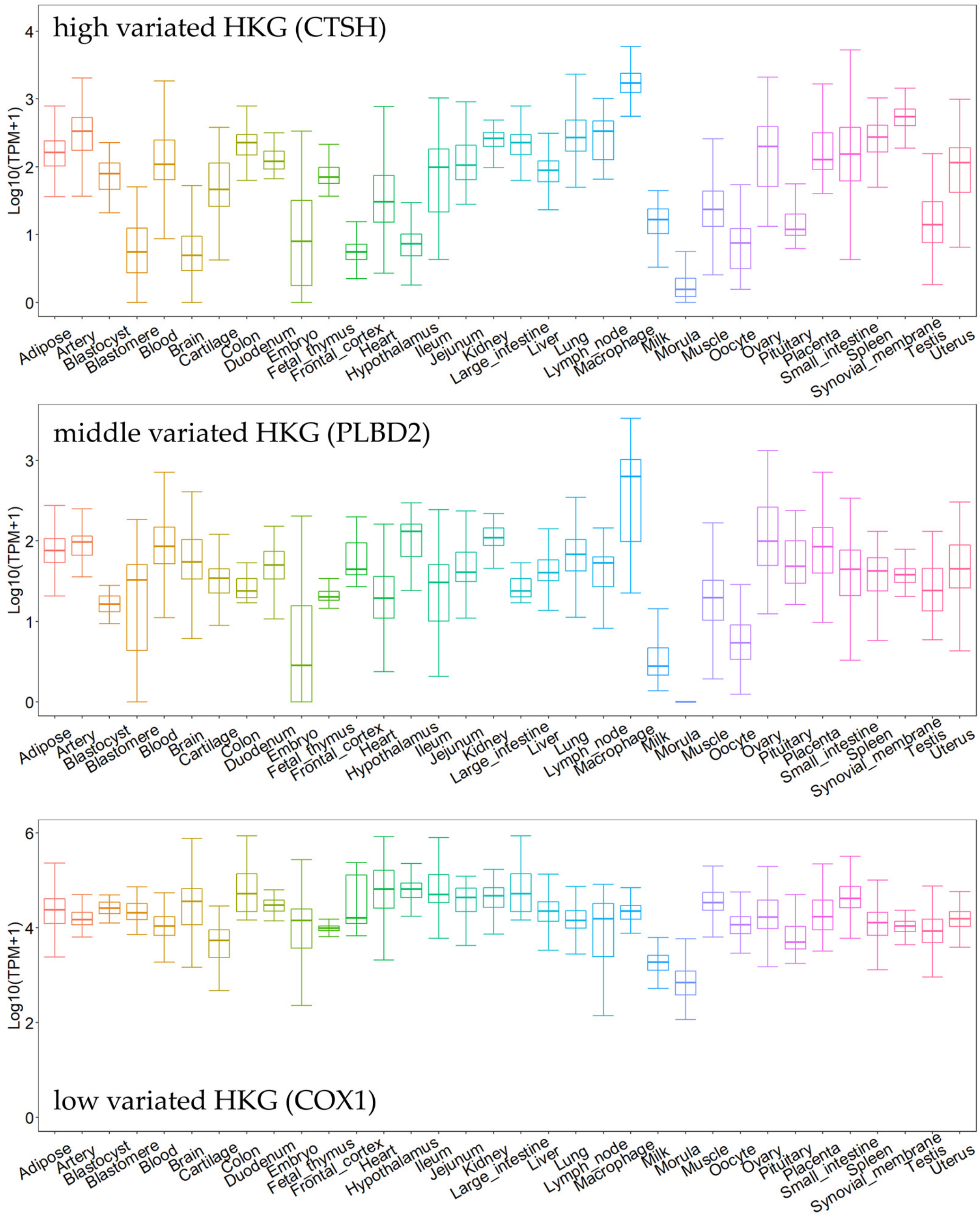

Figure S3. The expression of nine randomly selected HKGs in PigGTEx.

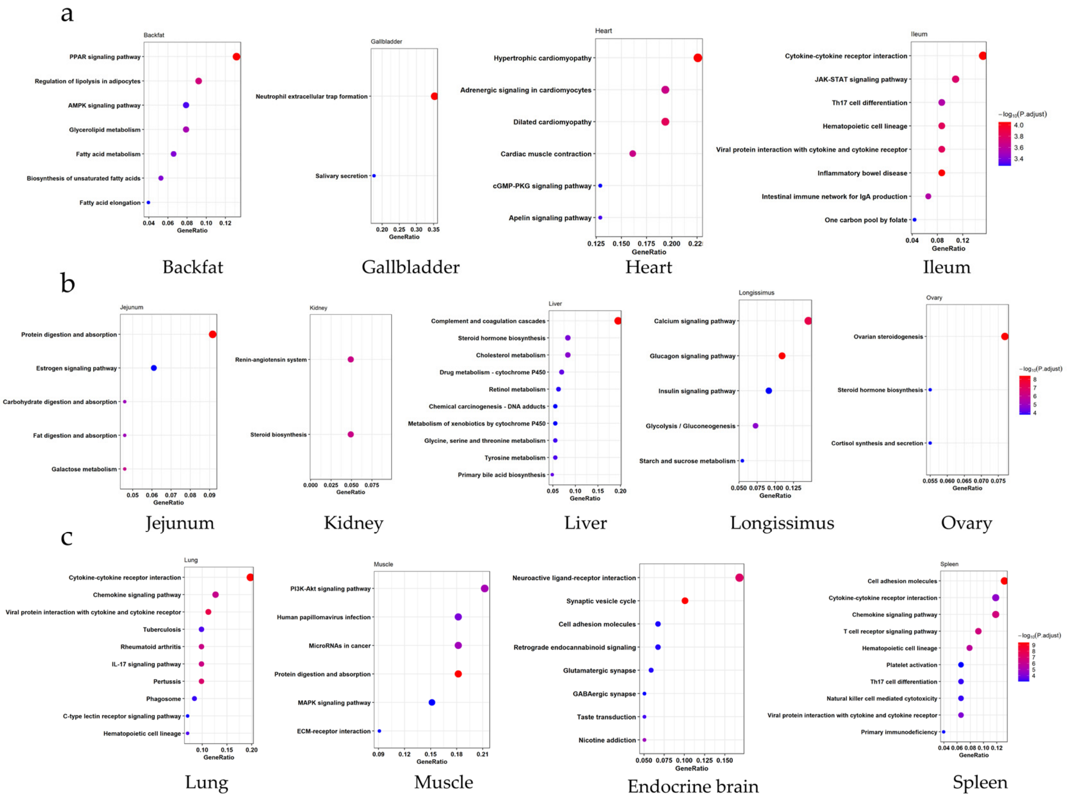

Figure S4. KEGG analysis of TSGs in each tissue. (a) KEGG in backfat, gallbladder, heart, and ileum; (b) KEGG in the jejunum, kidney, liver, longissimus, and Ovary; (c) KEGG in lung, muscle, pituitary, and spleen.

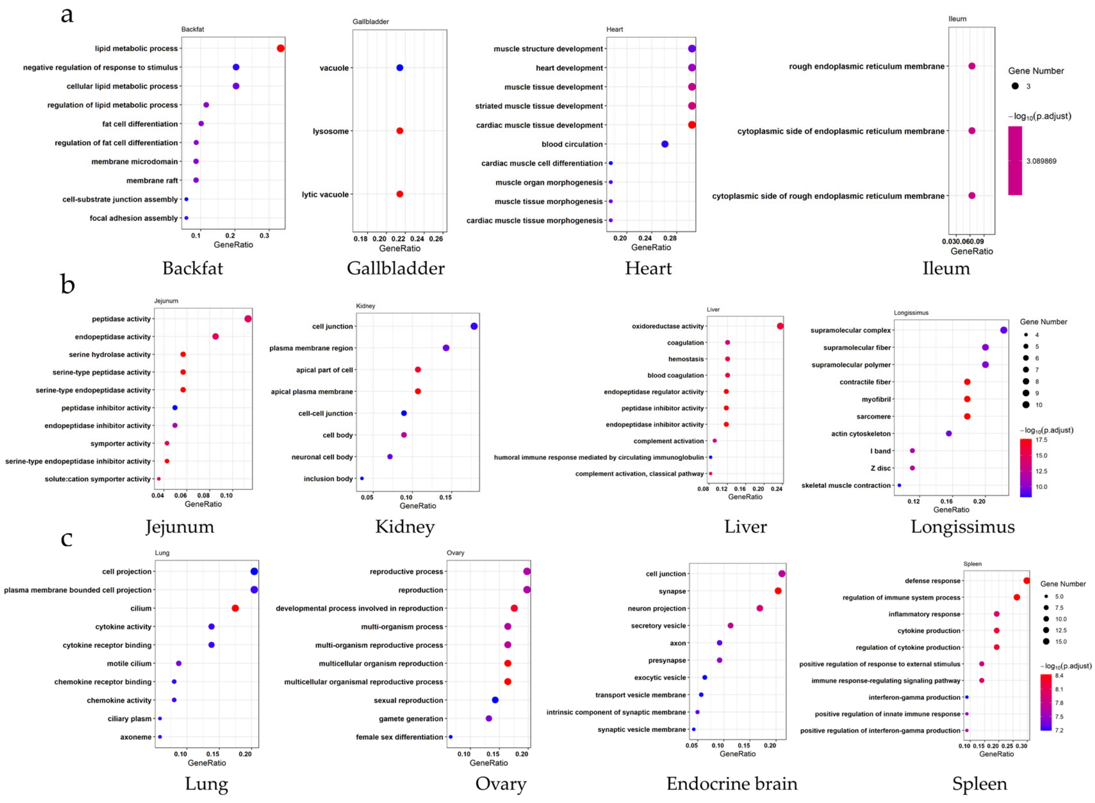

Figure S5. GO analysis of TSGs in each tissue. (a) GO in backfat, gallbladder, heart, ileum; (b) GO in the jejunum, kidney, liver, longissimus; (c) GO in lung, ovary, pituitary, spleen.

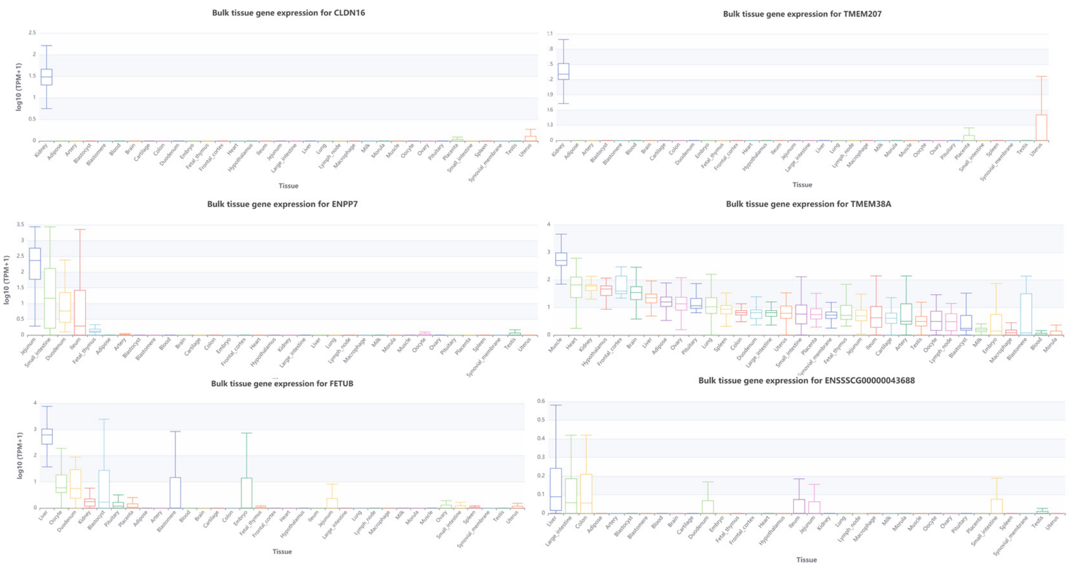

Figure S6. The expression of particular TSGs in PigGTEx.

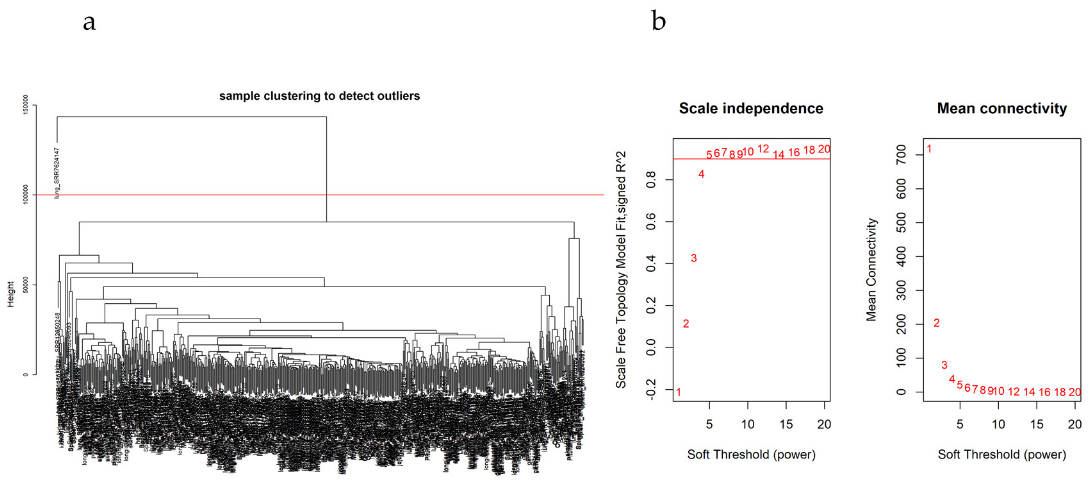

Figure S7. The weighted gene correlation network analysis. (WGCNA) in detail. (a) Hierarchical clustering of 14 tissues of pigs. (b) The determination of the power Beta (β) value.

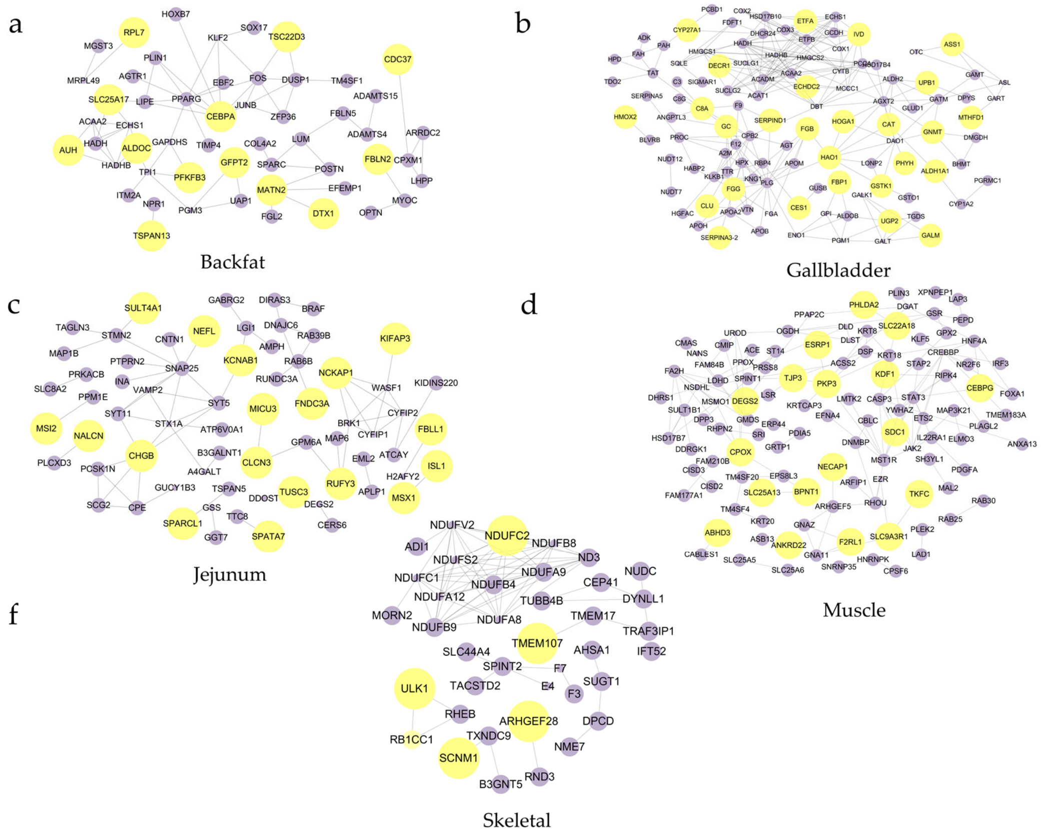

Figure S8. The network of interactions between genes highly correlated with traits including. (a) backfat; (b) gallbladder, (c) jejunum, (d) muscle, and (f) skeletal. The yellow represented HUGBs, and the purple represented non-HUGBs.
